# The Factors Associated With Decreasing Hemoglobin Levels and Platelet Counts After Trauma

**DOI:** 10.7759/cureus.55104

**Published:** 2024-02-27

**Authors:** Youichi Yanagawa, Hiroki Nagasawa, Soichiro Ota, Michika Hamada, Kenji Kawai, Hiroaki Taniguchi, Tatsuro Sakai, Hiromichi Ohsaka, Kazuhiko Omori

**Affiliations:** 1 Acute Critical Care Medicine, Juntendo University Shizuoka Hospital, Izunokuni, JPN

**Keywords:** inferior vena cava, fibrin degradation product, risk factor, platelet, hemoglobin

## Abstract

Objective

In this study, we investigated the factors related to anemia and platelet reduction in patients with moderate to severe trauma to gain a deeper understanding of these phenomena.

Methods

Our study spanned the period from April 2021 to September 2023, and it involved a retrospective review of the hospital medical charts of all emergency outpatients of all ages who were transported by a physician-staffed helicopter and treated at our hospital and were diagnosed with an Injury Severity Score (ISS) of >8 by CT on arrival. The following data were analyzed: sex; age; mechanism of injury; vital signs upon arrival at the hospital; ISS; hemoglobin level and platelet count on arrival and day two; fibrin degradation product (FDP) level, lactate dehydrogenase (LDH) level, and diameter of the inferior vena cava (IVC) on arrival; and infusion volume on day one. We then statistically calculated the independent risk factors for differences between hemoglobin levels and platelet counts on arrival and those on day two.

Results

The study included a total of 209 subjects, with an average age of 58 years and a male predominance. Multivariate analysis showed that the FDP level, IVC diameter, and age were significantly associated with changes in hemoglobin levels on arrival and day two, whereas the IVC diameter, LDH, age, systolic blood pressure, and sex were significantly associated with changes in the platelet count on arrival and day two.

Conclusions

A noteworthy correlation was found between certain factors and changes in hemoglobin levels and platelet counts between the initial assessment and the second day in our cohort. We recommend further prospective research to determine whether our findings hold true for a larger population of trauma patients.

## Introduction

In cases of trauma, bleeding often occurs, leading to the loss of red blood cells and consequently anemia [1,2]. Bleeding after trauma contributes to over half of the five million traumatic deaths reported every year [3,4]. In addition, the hemostatic mechanism is activated following trauma, causing a depletion of platelets and factors involved in coagulation [5]. In some cases, apart from surgical interventions at the site of injury or arterial embolization, replacement therapy may be required to address the reduction in these blood components. While previous studies have examined factors necessitating red blood cell and platelet transfusions after trauma, investigations into factors predicting the extent of anemia progression and platelet reduction following trauma have been scarce [6-8]. In light of this, we conducted this study to investigate the factors related to anemia and platelet reduction in patients with moderate to severe trauma to gain a deeper understanding of these phenomena.

## Materials and methods

We employed a retrospective design for this study and obtained approval from our institutional review board (approval number: 733, and the assessments adhered to the principles of good clinical practice as well as the guidelines outlined in the Declaration of Helsinki. A comprehensive retrospective analysis spanning the period between April 2021 and September 2023 was undertaken on the medical records of emergency outpatients of all age groups. These patients were transported by a physician-staffed helicopter and received care from emergency physicians in the Department of Acute Critical Care Medicine at our hospital. Specifically, we focused on those diagnosed with an Injury Severity Score (ISS) exceeding 8 based on CT scans upon arrival. The exclusion criteria included cases of mortality upon arrival, those requiring transfusion, or those needing major surgical procedures on the initial hospital day (day one). Notably, major operations excluded wound closure, external fixation, thoracostomy, or ventricular drainage.

Our facility functions as a base hospital without a designated trauma center and caters to the eastern region of Shizuoka, with an approximate population of 1,100,000. The annual influx of emergency room visits to our hospital totals around 13,000. Encompassing the Izu Peninsula, the combined area spans approximately 4,090 km² and features mountainous terrain. Due to the constraints of human medical resources, severely injured patients are primarily transported to our facility via physician-staffed helicopters or ground ambulances.

The analyzed dataset includes information such as sex, age, injury mechanism (blunt or penetrating), vital signs upon hospital arrival [Glasgow Coma Scale (GCS), systolic blood pressure, heart rate, and respiratory rate], ISS, hemoglobin levels on arrival and day two, platelet count on arrival and day two, fibrin degradation product (FDP) levels on arrival, lactate dehydrogenase (LDH) levels on arrival, the diameter of the inferior vena cava (IVC) at the suprarenal region on arrival, infusion volume on day one, and the final outcome (death or survival). Initial computations involved determining the variance in hemoglobin levels and platelet counts between arrival and day two. Subsequently, independent risk factors influencing these variances were statistically analyzed using multivariate logistic regression. Furthermore, a predictive formula for these variances was established based on the identified independent risk factors. We also applied the formula to the patients with regard to hemoglobin levels, who received transfusions but did not undergo surgery within the initial 24 hours of arrival.

Statistical analyses were executed using the JMP software program (version 15.0; SAS Japan Incorporation, Tokyo, Japan), with the threshold for statistical significance set at p<0.05. All presented data are expressed as mean ± standard deviation (SD), except for sex (number), GCS (median and interquartile range), and outcomes (number).

## Results

During the study period, 2,254 patients were transported by a physician-staffed helicopter. Among them, 266 patients had an ISS >8 due to trauma. After excluding 57 patients who had a cardiac arrest on arrival (n = 5) and required major operation and/or transfusion on day one (n = 52), the remaining 209 patients were enrolled as subjects. All cases, except one, which involved suicidal stab injury, were induced by blunt trauma. The characteristics of the subjects are presented in Table 1. The average age was 58 years, and the patients were predominantly male. The average hemoglobin level on day one was significantly greater than on day two (p<0.0001). The average platelet count on day one was also significantly greater than on day two (p<0.0001).

**Table 1:** Characteristics of the subjects (N=209) SD: standard deviation

Variables	Patient value	Unit
Age, mean ± SD	58.7 ± 20.6	Years
Sex, n	53/156	Female/male
Glasgow Coma Scale, median (interquartile range)	15 (14,15)	
Systolic blood pressure, mean ± SD	137.0 ± 28.2	mmHg
Heart rate, mean ± SD	92.4 ± 82.3	Beats per minute
Respiratory rate, mean ± SD	19.6 ± 4.6	Breaths per minute
Injury Severity Score, mean ± SD	14.2 ± 8.2	
Hemoglobin on day 1, mean ± SD	13.4 ± 1.7	g/dL
Hemoglobin on day 2, mean ± SD	11.7 ± 1.81	g/dL
Platelet on day 1, mean ± SD	230.5 ± 66.3	x × 10^3^/μL
Platelet on day 2, mean ± SD	186.6 ± 64.03	x × 10^3^/μL
Fibrin degradation products, mean ± SD	104.7 ± 158.2	μg/mL
Lactate dehydrogenase, mean ± SD	342.5 ± 123.7	IU/L
Inferior vena cava, mean ± SD	11.9 ± 4.8	mm
Infusion volume per day, mean ± SD	1958 ± 451	mL
Outcome, n	8/201	Death/survival

The multivariate analysis showed a significant association of the FDP level, IVC diameter, and age with differing levels of hemoglobin on arrival and day two (Table 2). The formula employed for determining the difference in the hemoglobin levels between arrival and day two was as follows: 2.69 + 0.002 × FDP - 0.05 × IVC - 0.01 × Age.

**Table 2:** Association with differing levels of hemoglobin on arrival and day two

Variables	Logworth	P-value
Fibrin degradation products	2.35	0.004
Inferior vena cava	2.13	0.007
Age	1.3	0.04
Sex	0.96	0.1
Glasgow Coma Scale	0.93	0.11
Infusion volume	0.57	0.26
Lactate dehydrogenase	0.43	0.37
Systolic blood pressure	0.38	0.4
Respiratory rate	0.37	0.42
Injury Severity Score	0.19	0.64
Heart rate	0.06	0.85

We applied the formula to the 40 patients regarding hemoglobin levels, who received transfusions but did not undergo surgery within the initial 24 hours of arrival. Since all 40 individuals received red blood cell transfusions, we investigated the correlation between the difference in values between the second day of illness and the day of admission calculated using the formula and the volume of red blood cell transfusions. As a result, the correlation coefficient was 0.01, indicating no correlation.

The multivariate analysis also showed a significant association of the IVC diameter, LDH level, age, systolic blood pressure, and sex with differing platelet counts on arrival and day two (Table 3). The formula used to determine the difference in the platelet count between arrival and day two was as follows: 81.35 - 2.09 × IVC + 0.05 × LDH - 0.30 × age - 0.18 × systolic blood pressure + sex [0.54 (male) or -0.54 (female)].

**Table 3:** Association with differing levels of platelet on arrival and day two

Variables	Logworth	P-value
Inferior vena cava	5.19	0.00001
Lactate dehydrogenase	2.26	0.005
Age	2.09	0.007
Systolic blood pressure	1.75	0.01
Sex	1.54	0.02
Fibrin degradation products	1.03	0.09
Glasgow Coma Scale	0.39	0.4
Respiratory rate	0.33	0.46
Infusion volume	0.31	0.48
Heart rate	0.16	0.67
Injury Severity Score	0.09	0.81

Since only three individuals received platelet transfusions, we excluded these three individuals and investigated the correlation coefficient between the difference in values between the second day of illness and the day of admission, and the value obtained from the formula, for the remaining 37 individuals. As a result, the correlation coefficient was 0.05, indicating no correlation.

## Discussion

This is the first study to propose formulae for predicting the difference in hemoglobin levels or platelet counts between arrival and day two. A noteworthy correlation was found between the FDP level, IVC diameter, and the patient's age and changes in hemoglobin levels between the initial assessment and the second day. A similarly substantial connection was found between the IVC diameter, LDH level, patient age and sex, and systolic blood pressure, and changes in the platelet count between the initial assessment and the second day.

In the current investigation, the factors linked to a decline in hemoglobin levels included FDP levels, IVC diameter, and age. Previous studies have highlighted the elevation of FDP levels in response to trauma, with a positive correlation observed between FDP levels and trauma severity [7,9]. The mechanism underlying this correlation may involve an upsurge in tissue factor release, leading to fibrinolysis or hematoma formation, depending on the severity of trauma [9]. Severe trauma can instigate substantial hemorrhaging, resulting in anemia. The IVC, a major vein supplying blood to the heart from the lower extremities and abdomen, serves as a potentially reliable indicator of hypovolemia in trauma patients based on CT findings [6,10]. Thinning or collapse of the IVC may signal hypovolemia due to trauma-induced hemorrhaging, progressing to shock, necessitating transfusion, and/or contributing to the onset of anemia [6,11]. Regarding age, prior research indicates that elderly trauma patients exhibit lower hemoglobin levels at admission, a higher frequency of transfusions, and consistently lower hemoglobin levels upon discharge compared to younger patients, when accounting for injury severity, comorbid conditions, and blood loss. The current study did not elucidate why increasing age seemed to impede the development of anemia post-trauma.

In this study, the factors associated with a reduction in platelet count included IVC diameter, LDH levels, age, systolic blood pressure, and sex. Concerning IVC diameter and age, it is speculated that a mechanism akin to that observed in anemia may be operative. Similar to IVC diameter, systolic blood pressure is presumed to reflect circulatory blood volume, suggesting that high systolic blood pressure may impede the reduction of hemoglobin levels post-trauma [8]. Regarding LDH, tissue damage leads to the release of LDH in direct proportion to the severity of the damage [12,13]. Similar to FDP, severe trauma can induce substantial hemorrhaging, resulting in thrombocytopenia. Lastly, a well-established phenomenon of a hypercoagulable state in women, evidenced by differences in blood coagulation studies between genders, was observed [14]. Female trauma patients are less likely to exhibit hyperfibrinolysis than males. Platelets from female patients demonstrate increased aggregation and fibrinogen receptor expression with adenosine diphosphate compared to male patients. This divergent biology appears to confer a theoretical protective effect for females after acute trauma, mitigating the risk of hemorrhaging. Thus, the female sex may have contributed to preventing thrombocytopenia after trauma in the current study.

There might be criticism as to why only some measures of underfilled vasculature, such as IVC diameter and systolic blood pressure, demonstrated correlation, while heart rate (which is a more sensitive marker compared to systolic blood pressure) and infusion volume (which provides direct evidence of underfilled vasculature) did not correlate. One possible reason for this is that, among the elderly, there may be a tendency toward bradycardia, or a lack of tachycardia response even in the presence of shock, due to factors such as age or the use of beta-blockers [15]. Given that the average age of our subjects was 58 years, the characteristics of the elderly may have influenced their heart rates, leading to the selection of blood pressure as a more sensitive indicator than heart rate. In addition, the volume of fluids administered may not have influenced the concentration of blood, as there are protocols that deliberately restrict fluid administration, especially in cases of hemorrhagic shock, and tolerate hypotension [16].

In this study, we attempted to apply the formula to cases of transfusion, but there was no clear correlation between the values obtained from the formula, the actual measured values, and the volume of transfusion. It could be explained by the relatively small size of the sample. Additionally, in cases where transfusions are administered (such as in cases of massive bleeding or active bleeding), factors such as fluid administration, ongoing bleeding tendencies, and the impact of transfusion itself on blood counts may affect the degree of decline compared to non-transfusion cases, suggesting the need to consider further factors.

Several limitations associated with this study merit acknowledgment. Firstly, it was a retrospective study with wide variability in certain data points, such as FDP levels, which, combined with the small sample size, may create the appearance of an association where none truly exists. Therefore, the data should be re-evaluated in a larger cohort. Secondly, patients with severe trauma who died upon arrival or required surgery or transfusion were not included in our analysis. Additionally, the exclusion of some cases with minor trauma (ISS <9) may have introduced selection bias. Furthermore, the derived formulae require validation in an independent cohort to be considered meaningful.

## Conclusions

In our study, a noteworthy correlation was found between certain factors and changes in hemoglobin levels and platelet counts between the initial assessment and the second day. We also established formulae for predicting the differences in the hemoglobin level and platelet count measured on the day of arrival versus those on day two.

## References

[1] Figueiredo S, Taconet C, Harrois A, Hamada S, Gauss T, Raux M, Duranteau J (2018). How useful are hemoglobin concentration and its variations to predict significant hemorrhage in the early phase of trauma? A multicentric cohort study. Ann Intensive Care.

[2] Kamiloski V, Kasapinova K (2017). Analysis of the hemoglobin level drop in patients with hip fracture after admission. Acta Clin Croat.

[3] Brohi K, Gruen RL, Holcomb JB (2019). Why are bleeding trauma patients still dying?. Intensive Care Med.

[4] Dobson GP, Morris JL, Letson HL (2022). Why are bleeding trauma patients still dying? Towards a systems hypothesis of trauma. Front Physiol.

[5] Pluta J, Trzebicki J (2019). Thrombocytopenia: the most frequent haemostatic disorder in the ICU. Anaesthesiol Intensive Ther.

[6] Chien CY, Yan JL, Han ST (2021). Inferior vena cava volume is an independent predictor of massive transfusion in patients with trauma. J Intensive Care Med.

[7] Lee DH, Lee BK, Noh SM, Cho YS (2018). High fibrin/fibrinogen degradation product to fibrinogen ratio is associated with 28-day mortality and massive transfusion in severe trauma. Eur J Trauma Emerg Surg.

[8] Carsetti A, Antolini R, Casarotta E (2023). Shock index as predictor of massive transfusion and mortality in patients with trauma: a systematic review and meta-analysis. Crit Care.

[9] Yanagawa Y, Ishikawa K, Jitsuiki K, Yoshizawa T, Oode Y, Omori K, Ohsaka H (2017). Fibrinogen degradation product levels on arrival for trauma patients requiring a transfusion even without head injury. World J Emerg Med.

[10] Ansari A, Kohar S, Dhok A, Onkar P, Mitra K, Ladke P (2022). Evaluation of normal inferior vena cava diameters in the Indian adult population by computed tomography. Cureus.

[11] Yanagawa Y, Sakamoto T (2008). Predicting the development of anemia by measuring the diameter of the inferior vena cava for children with blunt injuries. Pediatr Emerg Care.

[12] Jiang M, Qian H, Li Q, Han Y, Hu K (2023). Predictive value of lactate dehydrogenase combined with the abbreviated burn severity index for acute kidney injury and mortality in severe burn patients. Burns.

[13] Arslan G, Gemici AA, Yirgin IK, Gulsen E, Inci E (2013). Liver trauma grading and biochemistry tests. Emerg Radiol.

[14] Taghavi S, Tatum D, Reza T (2021). Sex differences in the massively transfused trauma patient. Shock.

[15] Zarzaur BL, Croce MA, Fischer PE, Magnotti LJ, Fabian TC (2008). New vitals after injury: shock index for the young and age x shock index for the old. J Surg Res.

[16] Tran A, Yates J, Lau A, Lampron J, Matar M (2018). Permissive hypotension versus conventional resuscitation strategies in adult trauma patients with hemorrhagic shock: a systematic review and meta-analysis of randomized controlled trials. J Trauma Acute Care Surg.

